# Awareness and diagnosis for intra-abdominal hypertension (IAH) and abdominal compartment syndrome (ACS) in neonatal (NICU) and pediatric intensive care units (PICU) – a follow-up multicenter survey

**DOI:** 10.1186/s12887-023-03881-x

**Published:** 2023-02-17

**Authors:** Paul Wiegandt, Thomas Jack, Alexander von Gise, Kathrin Seidemann, Martin Boehne, Harald Koeditz, Philipp Beerbaum, Michael Sasse, Torsten Kaussen

**Affiliations:** Department of Pediatric Cardiology and Intensive Care Medicine, Hannover Medical School, Carl-Neuberg-Street 1, 30625 Hannover, Germany

**Keywords:** Intraabdominal hypertension, Abdominal compartment syndrome, Survey, Newborn, Children, Neonatal intensive care, Paediatric intensive care

## Abstract

**Background:**

Constantly elevated intra-abdominal pressure (IAH) can lead to abdominal compartment syndrome (ACS), which is associated with organ dysfunction and even multiorgan failure. Our 2010 survey revealed an inconsistent acceptance of definitions and guidelines among pediatric intensivists regarding the diagnosis and treatment of IAH and ACS in Germany. This is the first survey to assess the impact of the updated guidelines on neonatal/pediatric intensive care units (NICU/PICU) in German-speaking countries after WSACS published those in 2013.

**Methods:**

We conducted a follow-up survey and sent 473 questionnaires to all 328 German-speaking pediatric hospitals. We compared our findings regarding awareness, diagnostics and therapy of IAH and ACS with the results of our 2010 survey.

**Results:**

The response rate was 48% (*n* = 156). The majority of respondents was from Germany (86%) and working in PICUs with mostly neonatal patients (53%).

The number of participants who stated that IAH and ACS play a role in their clinical practice rose from 44% in 2010 to 56% in 2016. Similar to the 2010 investigations, only a few neonatal/pediatric intensivists knew the correct WSACS definition of an IAH (4% vs 6%). Different from the previous study, the number of participants who correctly defined an ACS increased from 18 to 58% (*p* < 0,001). The number of respondents measuring intra-abdominal pressure (IAP) increased from 20 to 43% (*p* < 0,001). Decompressive laparotomies (DLs) were performed more frequently than in 2010 (36% vs. 19%, *p* < 0,001), and the reported survival rate was higher when a DL was used (85% ± 17% vs. 40 ± 34%).

**Conclusions:**

Our follow-up survey of neonatal/pediatric intensivists showed an improvement in the awareness and knowledge of valid definitions of ACS. Moreover, there has been an increase in the number of physicians measuring IAP in patients. However, a significant number has still never diagnosed IAH/ACS, and more than half of the respondents have never measured IAP. This reinforces the suspicion that IAH and ACS are only slowly coming into the focus of neonatal/pediatric intensivists in German-speaking pediatric hospitals. The goal should be to raise awareness of IAH and ACS through education and training and to establish diagnostic algorithms, especially for pediatric patients.

The increased survival rate after conducting a prompt DL consolidates the impression that the probability of survival can be increased by timely surgical decompression in the case of full-blown ACS.

**Supplementary Information:**

The online version contains supplementary material available at 10.1186/s12887-023-03881-x.

## Contributor’s statement page

All above mentioned authors meet the following criteria:

1. Substantial contributions to conception and design, acquisition of data, analysis and interpretation of data.
2. Drafting the article or revising it critically for important intellectual content; and
3. Final approval of the version to be published.

## Background

In neonatal (NICU) and pediatric intensive care units (PICUs), abdominal compartment syndrome (ACS) is a poorly recognized condition where prolonged elevated intra-abdominal pressure (IAP) can lead to multiorgan-dysfunction or even progress to multiorgan failure [1].

Our 2010 survey revealed an inconsistent acceptance of definitions and guidelines regarding the diagnosis and treatment of intra-abdominal hypertension (IAH) and ACS among neonatal/pediatric intensive care physicians [2].

In the meantime (2013), the Abdominal Compartment Society (WSACS) has published updated definitions and guidelines regarding the diagnosis and treatment of intra-abdominal hypertension (IAH) and ACS in children and adolescents [3]. IAH exists when the IAP is ≥10 mmHg in 2 consecutive measurements. One speaks of an ACS when new or aggravated organ dysfunctions manifest themselves under IAH.

To assess the impact of the updated WSACS guidelines, we sent a new questionnaire to all German-speaking NICU/PICU directors in 2016. This paper aims to show an overview of the situation in D-A-CH (Germany, Austria, Switzerland) regarding the awareness and treatment of IAH and ACS in PICUs.

We assumed that there has been an increase in awareness of IAH and ACS in pediatric patients as well as an uptake of unified measurement and guideline-based therapy concepts since 2010.

## Methods

In May 2016, 473 questionnaires were sent to all 328 heads of neonatal and pediatric intensive care units (NICU or PICU) in Germany, Austria, and Switzerland (D-A-CH). For this purpose, hospital and department lists of the respective national specialist societies were analyzed and all heads of the respective department or intensive care unit were contacted directly. In order to detect possible differences in response behaviour, the participants were asked whether their intensive care units (ICU) tended to care for premature and newborn babies (= “NICU”; responsible up to the age of 28 days), or rather older children from infancy to adolescence (= “PICU”; responsible for babies and children beyond the age of 28 days). The vast majority of German-speaking ICUs in pediatric hospitals represent mixed forms with a predominant “NICU” share (see Table 2 + Suppl. II). Questionnaires were personally addressed to ICU directors with a request for return within 8 weeks. After 4 weeks, a “reminder e-mail” was sent.

The questionnaire included 4 questions on department/clinic description and 6 sets on IAH/ACS to be answered either by multiple-choice or text (see Appendix 1 for the questionnaire).

Inclusion criteria: Answer sheets should be returned within 8 weeks via post, fax or mail. Questionnaires were pseudonymized and digitally coded upon return. Completed questionnaires were compared with incomplete questionnaires.

No exclusion criteria beyond the opposite of the inclusion criteria were defined.

Extracts from the 2010 questionnaire survey results were published in Annals of Intensive Care 2012 (excluding the Austrian and Swiss results). In order to be able to directly compare the dynamics of the development of awareness and diagnosis with the results of the 2010 survey, the questionnaire was left virtually unchanged and only the following questions were added:

- “Did you participate in the 2010 survey?"- "In 2015, how many times did your hospital have to leave the abdomen temporarily open postoperatively?”

For this reason, the reliability and validity of the questionnaire was not re-examined.. The theoretical basis of the questionnaire made use of the definitions and therapeutic options published by WSACS in 2013. The questionnaire can be found in the supplement of this article (translated into English).

The survey administration and analysis were self-funded by the authors.

Results were entered into a custom-designed database using Office Excel 2016 for Mac (Microsoft®, Redmond, Washington, USA), analyzed descriptively, and reported in rounded absolute numbers or percentages. The chi-square test for independent samples was used to statistically represent differences between the 2010 and 2016 results. The PSPP version 1.0.1. for Mac (GNU Project, Boston, USA) was used to analyze the data.

## Results

Four hundred seventy-three questionnaires were sent to 328 heads of NICU’s and/or PICU’s in Germany, Austria, and Switzerland (D-A-CH). The majority of respondents came from Germany (Table 1).

**Table 1:** Respondent demographics. Table compares the demographics of respondents in 2010 and 2016

Country	2010 *n* = 155	2016 *n* = 156
Germany (D)	82% (127)	86% (134)
Austria (A)	11% (17)	5% (8)
Switzerland (CH)	7% (11)	9% (14)

Large hospitals with multiple neonatal and/or pediatric intensive care units returned one sheet (representative of all ICUs). In total, the response rate was 48% (156/328). Table 2 shows the structure and specialization of the participating PICUs: In comparison to the 2010 survey, the distribution of the hospitals responding has remained about the same (Table 2).

**Table 2:** Descriptive statistics concerning the structure of answering clinics. Table compares descriptive statistics of hospitals responding in 2010 and 2016

Factor	Structure and orientation of ICU	2010	2016
Participation in the 2010 survey			*n* = 152 97%
Medical focus of the ICU		*n* = 155	*n* = 156
	Exclusive NICU	17%	26%
	NICU rather than PICU	53%	56%
	PICU rather than NICU	12%	8%
	Exclusive PICU	4%	5%
	Not specified	14%	5%
Age distribution of patients treated		*n* = 155	*n* = 156
	Neonatologic	70%	73%
	Pediatric	30%	27%
Level of medical care at participating NICU’s		*n* = 148	*n* = 156
	High level	53%	72%
	Intermediate level	17%	13%
	Low level	12%	15%
	Not specified	18%	
Size of ICU/ Number of cases in 2009/2015		*n* = 155	*n* = 156
	< 351 patients/year	30%	35%
	351 to 700 patients/year	33%	32%
	> 700 patients/year	23%	19%
Part of university hospitals		n = 155	n = 156
		30%	31%

*ICU* Intensive care unit; *NICU* neonatal intensive care unit (for premature and newborn infants (up to 28 days of life)); *PICU* pediatric intensive care unit (for older children from infancy to adolescence (beyond the 28th day of life))

### Awareness of IAH und ACS

The majority of all ICUs stated that IAH and ACS are present in everyday clinical practice.

**Fig. 1:**
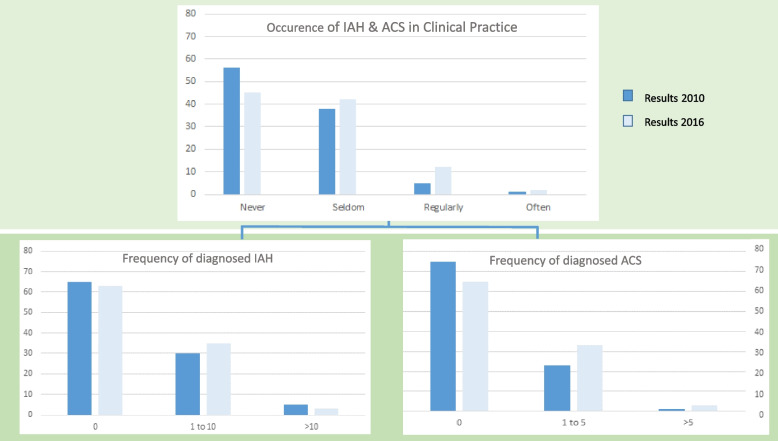
Occurrence of IAH and ACS in clinical practice

Compared to the 2010 survey, more physicians (44% vs. 55%) reported that IAH and ACS play a role in clinical practice (Fig. 1; B.1 of Table 3), and 13% of respondents reported even a more frequent diagnosis of IAH and ACS since 2010 (B.2 of Table 3). The diagnosis of IAH was made as often by respondents in 2010 as in 2016, whereas ICUs diagnosing at least one case of ACS increased from 25 to 35% (B.3 of Table 3).

**Table 3:** Distribution of responses. Table shows the responses given by the pediatric intensive care units surveyed regarding awareness, diagnosis, and treatment of IAH and ACS between 2010 and 2016 (subjective, semi-quantitative assessment). A chi-square test for independent samples was used to statistically represent differences between 2010 and 2016

Question	Stated question and choices	2010 Answers % (number)	2016 Answers % (number)	***p***-value
B.1	Occurrence and relevance of IAH/ACS in clinical practice			0.088
	• Never	56 (83/149)	45 (69/155)	
	• Seldom	38 (57/149)	42 (65/155)	
	• Regularly	5 (8/149)	12 (18/155)	
	• Often	1 (1/149)	2 (3/155)	
B.2	Increase in diagnosis of IAH and ACS since 2010		13 (21/156)	
B.3	Frequency of IAH diagnosed at ICU’s responding (during the entire year before)			0.366
	• 0 times IAH	65 (96/148)	63 (94/150)	0.130
	• 1 to 10 times IAH	30 (44/148)	35 (52/150)	
	• > 10 times IAH	5 (8/148)	3 (4/150)	
	Frequency of ACS diagnosed at ICU’s responding (during the entire year before)			
	• 0 times ACS	75 (112/149)	65 (95/147)	
	• 1 to 5 times ACS	23 (35/149)	33 (48/147)	
	• > 5 times ACS	1 (2/149)	3 (4/147)	
B.4	Awareness and use of current WSACS definitions (tested by multiple choice)			
	• IAH definition correctly chosen (increased IAP)	4 (6/147)	6 (6/97)	0.457
	• ACS definition correctly chosen (IAH + new organ dysfunction)	18 (27/147)	58 (58/99)	**< 0.001**
	Clinical symptoms stated to be associated with increased IAP in children (Fig. 3)			
	Respiratory-organ-systems:			
	Respiratory insufficiency	13 (25/195)	9 (25/283)	
	Radiologic findings with diaphragmatic protrusion	8 (16/195)	13 (38/283)	
	Cardiovascular-organ-systems:			
	Perfusion deficit	8 (16/195)	6 (16/283)	
	Cardio-circulatory insufficiency	4 (8/195)	7 (21/283)	
	Impaired venous reflux, increased CVP	4 (7/195)	7 (19/283)	
	Lactic acidosis	1 (1/195)	1 (3/283)	
	Shock and capillary leak syndrome	1 (1/195)	1 (2/283)	
	Renal-organ-system:			
	Oliguria to anuria	21 (40/195)	21 (60/283)	
	Gastrointestinal-organ-system:			
	Clinical abdominal symptoms, pain	29 (57/195)	29 (83/283)	
	Gastrointestinal motility dysfunction	5 (10/195)	4 (10/283)	
	Hepatic-organ-system:			
	Liver insufficiency and ascites	2 (4/195)	1 (4/283)	
	Others:			
	No further differentiated organ dysfunction	5 (9/195)	1 (2/283)	
	Anamnesis	1 (1/195)	n/a	
B.5	Share of respondents who measured IAP			
	• Yes	20 (30/151)	43 (65/152)	**< 0,001**
	○ Seldom		29 (44/152)	
	○ Regularly	20 (30/151)	11 (17/152)	
	○ Often		3 (4/152)	
	• No	80 (121/151)	57 (87/152)	
B.6	Share of respondents having performed at least one decompressive laparotomy	19 (29/149)	36 (55/153)	**< 0,001**
	Stated survival rate of ACS patients			0,84
	• Surgically treated children	83 ± 32 (*n* = 23)	85 ± 17 (*n* = 48)	
	• Non- surgically treated children	65 ± 44 (*n* = 10)	40 ± 34 (*n* = 33)	
	Share of respondents who needed to leave the abdomen open postoperatively (only 2016)		95 (39/41)	

*Abbrev*.: *ACS* Abdominal compartment syndrome, *CVP* Central venous pressure, *IAH* Intra-abdominal hypertension, *IAP* Intra-abdominal pressure, *ICU* Intensive care unit

The *p* values are taken from the analysis using the chi square test

Survey participants were asked whether IAH and ACS play a role in their daily clinical practice. The results were compared with the preliminary findings from the 2010 survey.

### Definition and diagnosis of IAH and ACS

The knowledge of the correct definition of IAH remains low among all physicians who responded. Only 4% in 2010 and 6% in 2016 chose the correct answer. In contrast, the number of ICUs that knew how to define an ACS in accordance with the updated WSACS guidelines increased from 18% in 2010 to 58% in 2016 (*p* < 0,001, B.4 in Table 3).

**Fig. 2:**
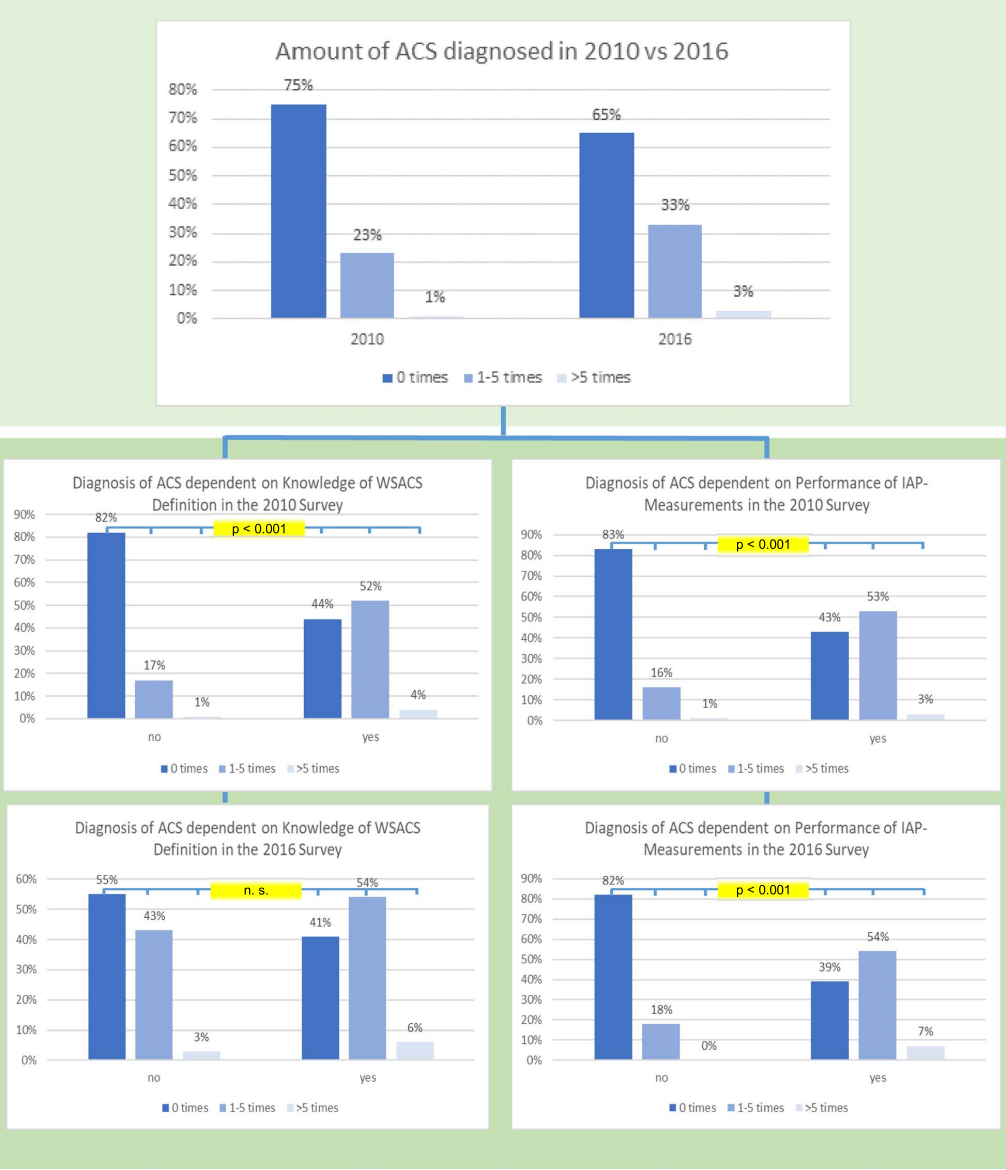
Reported cases of ACS dependent on knowledge of valid WSACS definitions and performance of IAP measurements

However, in 2016, a large proportion of clinics responding still diagnosed IAH (50%, 49/97) and ACS (40%, 40/99) exclusively by using clinical symptoms. In contrast, our results could show that the diagnosis and perception of ACS increases when the valid definitions are known and the IAP is measured (Fig. 2).

Respondents were asked to indicate how many cases of ACS had been diagnosed in the previous year. We analyzed the results considering the knowledge of the valid WSACS definitions and regular IAP measurements. The results were compared with the primary survey from 2010. The *p*-value refers to the agregration of all three response options.

**Fig. 3:**
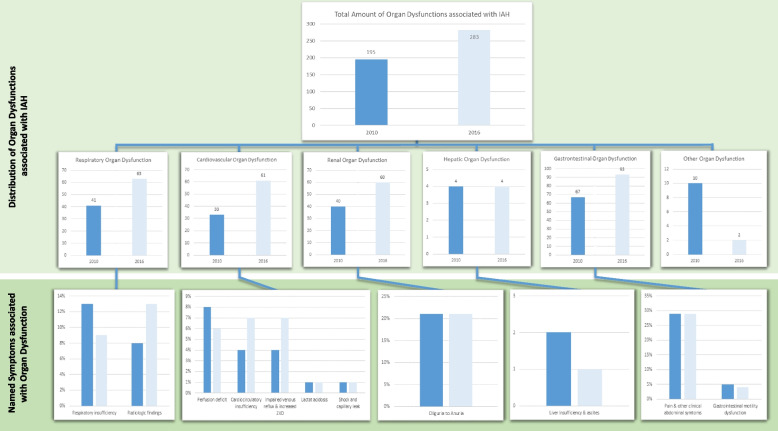
Clinical symptoms stated as associated with increased IAP in children

When subjects were asked about clinical symptoms in pediatric patients associated with elevated IAP, clinical abdominal symptoms emerged as the most common symptom in both 2010 (29%) and 2016 (29%) (B.4 in Table 3). An overview of organ dysfunctions associated with elevated IAP in children is shown in Fig. 3.

Respondents were asked to name three clinical symptoms associated with increased intra-abdominal pressure (multiple answers possible). The symptoms were sorted based on the Pediatric Organ Dysfunction Information Update Mandate (PODIUM) [4]. A chi-square test for independent samples was used to statistically represent differences from 2010 to 2016.

### Measurement of IAP

The number of clinics measuring IAP almost doubled in 2016 compared to 2010 (20% vs. 43%, *p* < 0,001).

However, the majority of those measuring IAP stated that they seldom measured IAP (29%). Only 3% of respondents measured IAP regularly (B.5 in Table 3).

### Therapeutical strategies concerning the management of IAH/ ACS patients

The share of respondents using decompressive laparotomies (DLs) to reduce IAP increased from 19 to 36% (*p* < 0,001, B.6 in Table 3). When asked about the survival rate of ACS in both 2010 and 2016, they stated that it was higher if the patient was treated surgically. 39 of 41 (95%) respondents reported having left the abdomen open postoperatively after a DL.

## Discussion

Our results came from the first nationwide survey of neonatal/pediatric intensive care units in German-speaking countries (D-A-CH) after the updated WSACS guidelines for pediatric patients were published in 2013. We compared our findings regarding awareness, diagnostics, and therapy of IAH and ACS with the results of our survey from 2010.

### Literature overview

No more than one other survey among pediatric health care providers has been published since the WSACS guidelines were issued in 2013. We compared our findings regarding awareness and intra-abdominal pressure measurement with those of Rezeni et al., who conducted a survey among all physicians in PICUs in Saudi Arabia in 2022 and with those of Newcombe et al., who published their follow up survey of pediatric nurses in 2012, before WSACS released its updated guidelines [1, 5].

Due to the small number of exclusively pediatric surveys, we additionally compared our results with large, international surveys where at least some of the respondents were pediatricians [6, 7].

### Awareness of IAH and ACS

In our survey, there was an improvement in awareness of IAH and ACS between 2010 and 2016. Consistent with our findings, Newcombe also showed an increase in their survey of pediatric nurses regarding awareness of ACS; however, theirs was in a much higher percent range (69% vs. 88%) [1].

The number of responding ICUs who never experienced an ACS in their clinical practice (2016: 45%) and those who never diagnosed ACS (2016: 65%) confirms the suspicion that the valid definitions provided by WSACS regarding correct diagnosis and IAP monitoring had not yet reached all pediatric hospitals in 2016.

### Definition and diagnosis of IAH and ACS

During the 6-year follow-up period, the proportion of ICUs correctly defining an ACS rose to 58%. In contrast both, the Newcombe survey, where the percentage of participants who knew the correct ACS definition decreased (19.5 to 13.2%) and the Rezeni survey, where only 10% of participants knew the correct definition of an ACS our survey shows a positive trend regarding the correct diagnosis of ACS in German-speaking countries [1, 5].

Unfortunately, the present survey results give the impression that many cases of IAH and ACS are being missed because respondents ignore the valid definitions or even do not know them. Commensurate to our results, Wise et al. proved that participants aware of the WSACS definition also identified more cases of ACS [7]. Critically, in 2016, those with knowledge of the valid WSACS definition did not diagnose significantly more cases of ACS. The definitions and basic knowledge supporting these procedures in an evidence-based manner must reach a certain level of penetration before new methods find their way into everyday clinical practice. Fittingly, there was a greater increase in respondents who knew the valid WSACS definition (+ 40%) than in those who measured IAP (+ 23%).

### Measurement of IAP

In both the 2010 and 2016 surveys, more than half of the respondents said they did not measure IAP. In Newcombe’s survey, only one quarter of respondents said they did not measure IAP, while in Rezeni’s survey only one third did not measure the IAP [1, 5].

Measuring IAP is the only reliable method of detecting intrabdominal pressure and is essential for further treatment planning. The high number of respondents diagnosing ACS based solely on clinical symptoms (40%) suggests that even in 2016 many intensivists incorrectly rely on clinical examination to assess IAP. This is consistent with the survey results of Wise et al., where 18% of respondents never measured IAP, only relying on clinical examination [6]. Rezeni’s survey results also showed, that 45% incorrectly relied on clinical examination [5].

Several research groups have demonstrated that clinical assessment of the abdomen is not adequate to quantify IAP and is only associated with about 50% sensitivity [8, 9].

In the 2010 survey, we showed that most participants would measure IAP if measurement methods were easier to integrate into everyday clinical practice. The validation study of our research group could show that intragastric pressure measurement reflects IAP well and requires significantly less measurement effort [10].

### Therapeutical strategies concerning the management of IAH/ ACS patients

The therapeutical goal for IAH is to prevent a progression to ACS. Several non-surgical therapeutic strategies are recommended by WSACS to reduce elevated IAP before surgical treatment is necessary [3]:

1. Evacuation of intraluminal contents;
2. Evacuation of intraabdominal space occupying lesions;
3. Improvement of abdominal wall compliance;
4. Optimization of fluid administration; and.
5. Optimization of systemic and regional perfusion.

Jacobs et al. demonstrated in their review the importance of considering fluid balance in the management of IAH and ACS [11]. Rezeni provided an overview of the knowledge and prevalence of the use of conservative non-invasive treatment options [5].

When conservative therapy for ACS fails, the indication for a DL is given. If an ACS is left untreated, this results in a mortality rate of up to 100% [12]. Recently a pediatric study of di Natale et al. demonstrated the limitation of DLs in children with ACS: Although correctly diagnosed and treated, ACS had a high mortality rate of 57% [13]. This again underlines the importance of measuring IAP in critically ill children and performing a DL immediately if an ACS is detected.

Known as a therapeutic maximum treatment option in fulminant ACS, a DL was used almost twice as often in 2016 as in 2010. In 2019, Wise et al. showed that a DL is the most commonly used therapeutic option in the management of IAH and ACS. However, pediatricians or pediatric surgeons were the least likely to use this therapeutic option compared with other disciplines [7]. Thus, it would be reasonable to assume that the inhibition threshold to DL seems to be higher in pediatric patients with ACS than in adults.

Consistent with the results of Strang et al., both 2010 and 2016 surveys showed a significantly higher likelihood of survival when a DL was performed (B.6 of Table 3) [14].

### Prognosis of children suffering from ACS

In both surveys, we were able to show that the probability of survival can be increased by timely surgical decompression in the case of a full-blown ACS. In his retrospective work, DeWaele was able to show that there seems to be an average of 18 hours between diagnosis of ACS and a DL in adults [15].

The time latency between IAP elevation, clinical deterioration, time of transition to ACS, and the subsequent decision to perform a DL is crucial for the prognosis. Silveira et al. demonstrated that even a low level of IAH in children can lead to cardiac dysfunction [16].

Furthermore, as shown in Table 2, elevated IAP can also lead to dysfunction of many other organ systems (up to and including multi-organ failure). The research group around Agyeman could show that mortality increases dramatically with an increasing number of organ dysfunctions [17].

### Dependence of questionnaire response behaviour on influencing factors

Knowledge and implementation of the definitions of IAH and ACS seemed to be independent of the medical orientation of the respective ICU and the experience or familiarity of the ICU physicians with these two disease entities (see Suppl. II + III). With regard to all other survey questions, on the other hand, it was found that the relevance of IAH/ACS was assessed more highly in paediatric patients rather than in neonatology patients, that the diagnoses were considered more frequently and had been made more often since 2010, and that decompressive laparotomies were performed more regularly. At the same time, there was a correlation between familiarity with both entities and the completeness of returned questionnaires. These knowledge and application advantages of PICUs over NICUs were already apparent in 2010; although detailed knowledge has increased on average in all ICUs since 2010, PICUs seem to have tended to learn faster and more in recent years (see Suppl. II + III).

#### Limitation of this survey

Due to the 48% response rate, our survey represents only a part of the pediatric intensive care units in German-speaking countries. Moreover, some questionnaires were only partially completed, thus reducing the validity of the survey. As in every survey, the questionnaires returned reflect the subjective experience of pediatric intensivists regarding IAH and ACS and are not objective data. Despite the limitations mentioned, we would have expected a higher number of diagnosed IAH and ACS cases as well as a larger proportion of pediatricians measuring IAP. The results do not reflect the current status because there was a relevant delay between assessment and the reporting of this dataset, which was solely due to a lack of resources.

## Conclusion

This is the first survey of neonatal/pediatric intensivists to assess the impact of the updated guidelines published by WSACS in 2013. Our study showed increasing awareness and a higher number of respondents knowing the correct definition of an ACS. Despite this, a large number of respondents stated that they had never diagnosed IAH/ACS or measured IAP. Future aspects should include regular training and education for pediatric clinicians and nurses to increase awareness of IAH and ACS and, thus, improve knowledge of diagnostics and therapy.

Our data also showed an improvement in the survival rate if a DL is performed in a timely manner.

It is necessary to establish evidence-based therapy algorithms to reduce the inhibition threshold to invasive interventions in the context of timely ACS treatment.

## Supplementary Information

**Additional file 1.** Supplement: survey questions.

**Additional file 2: Supplement II.** Presentation of answers depending on the medical focus of the intensive care units (ICU).

**Additional file 3: Supplement III.** Presentation of answers depending on complete versus incomplete questionnaires (2016 survey).

## Data Availability

Raw data and calculations can be obtained from T.K., P.W. or from the secretariat of the Department of Paediatric Cardiology and Intensive Care Medicine at the University Children’s Hospital, Hannover Medical School.

## Abbreviations

A: Austria
ACS: Abdominal Compartment Syndrome
CH: Switzerland
CVP: Central venous pressure
D: Germany
DL: Decompressive laparotomy
IAH: Intra-abdominal hypertension
IAP: Intra-abdominal pressure
ICU: Intensive Care Unit
MHH: Hannover Medical School (in German Medizinische Hochschule Hannover)
n: number
NICU: Neonatal intensive Care Unit
PICU: Pediatric Intensive Care Unit
PODIUM: Pediatric Organ Dysfunction Information Update Mandate
WSACS: Abdominal Compartment Society (formerly “World Society of ACS”)

## References

[1] Newcombe J, Mathur M, Bahjri K, Ejike JC (2012). Pediatric critical care nurses’ experience with abdominal compartment syndrome. Ann Intensive Care.

[2] Kaussen T, Steinau G, Srinivasan PK, Otto J, Sasse M, Staudt F (2012). Recognition and management of abdominal compartment syndrome among German pediatric intensivists: results of a national survey. Ann Intensive Care.

[3] Kirkpatrick AW, Roberts DJ, De Waele J, Jaeschke R, Malbrain MLNG, De Keulenaer B (2013). Intra-abdominal hypertension and the abdominal compartment syndrome: updated consensus definitions and clinical practice guidelines from the world Society of the Abdominal Compartment Syndrome. Intensive Care Med.

[4] Bembea MM, Agus M, Akcan-Arikan A, Alexander P, Basu R, Bennett TD (2022). Pediatric organ dysfunction information update mandate (PODIUM) contemporary organ dysfunction criteria: executive summary. Pediatrics.

[5] Rezeni N, Thabet F (2022). Awareness and management of intra-abdominal hypertension and abdominal compartment syndrome by paediatric intensive care physicians: a national survey. Anaesthesiol Intensive Ther.

[6] Wise R, Rodseth R, Blaser A, Roberts D, De Waele J, Kirkpatrick A (2019). Awareness and knowledge of intra-abdominal hypertension and abdominal compartment syndrome: results of a repeat, international, cross-sectional survey. Anaesthesiol Intensive Ther.

[7] Wise R, Roberts DJ, Vandervelden S, Debergh D, De Waele JJ, De Laet I (2015). Awareness and knowledge of intra-abdominal hypertension and abdominal compartment syndrome: results of an international survey. Anaesthesiol Intensive Ther.

[8] Kirkpatrick AW, Brenneman FD, McLean RF, Rapanos T, Boulanger BR (2000). Is clinical examination an accurate indicator of raised intra-abdominal pressure in critically injured patients?. Can J Surg.

[9] Sugrue M, Bauman A, Jones F, Bishop G, Flabouris A, Parr M (2002). Clinical examination is an inaccurate predictor of intraabdominal pressure. World J Surg.

[10] Kaussen T, Gutting M, Lasch F, Boethig D, von Gise A, Dingemann J (2021). Continuous intra-gastral monitoring of intra-abdominal pressure in critically ill children: a validation study. Intensive Care Med Exp.

[11] Jacobs R, Wise RD, Myatchin I, Vanhonacker D, Minini A, Mekeirele M (2022). Fluid management, intra-abdominal hypertension and the abdominal compartment syndrome: a narrative review. Life (Basel).

[12] Ejike JC, Humbert S, Bahjri K, Mathur M (2007). Outcomes of children with abdominal compartment syndrome. Acta Clin Belg.

[13] di Natale A, Moehrlen U, Neeser HR, Zweifel N, Meuli M, Mauracher AA (2020). Abdominal compartment syndrome and decompressive laparotomy in children: a 9-year single-center experience. Pediatr Surg Int.

[14] Strang SG, Van Lieshout EM, Verhoeven RA, Van Waes OJ, Verhofstad MH, IAH-ACS Study Group (2017). Recognition and management of intra-abdominal hypertension and abdominal compartment syndrome; a survey among Dutch surgeons. Eur J Trauma Emerg Surg.

[15] De Waele JJ, Hoste EA, Malbrain ML (2006). Decompressive laparotomy for abdominal compartment syndrome--a critical analysis. Crit Care.

[16] Silveira LGT, Brocca IC, Moraes ES, Brandao MB, Nogueira RJN, de Souza TH (2021). Hemodynamic effects of increased intra-abdominal pressure in critically ill children. J Pediatr.

[17] Agyeman PKA, Schlapbach LJ, Giannoni E, Stocker M, Posfay-Barbe KM, Heininger U (2017). Epidemiology of blood culture-proven bacterial sepsis in children in Switzerland: a population-based cohort study. Lancet Child Adolesc Health.

