# Supplementary material for: Awareness and diagnosis for intra-abdominal hypertension (IAH) and abdominal compartment syndrome (ACS) in neonatal (NICU) and pediatric intensive care units (PICU) – a follow-up multicenter survey
Source: BMC Pediatr. 2023 Feb 17;23:82. doi: 10.1186/s12887-023-03881-x (PMC9936744; doi:10.1186/s12887-023-03881-x)
Supplement: Supplementary file 1 — Additional file 1. Supplement: survey questions. [file 12887_2023_3881_MOESM1_ESM.docx]

**Supplement: Survey Questions**

**Part A: Description of the clinics**

A.1 Did you participate in our first survey in 2010?

- Yes
- No

A.2 Your clinic/department is:

- Only NICU
- Only PICU
- NICU>PICU
- PICU>NICU

If NICU, then

- Level 1
- Level 2
- Level 3

A.3 Size of ICU/ Number of cases in 2015:

- <350
- 351-700
- >700

Including neonatological _______ (%)

Including pediatric _______ (%)

A.4 The mission of your clinic/department is: (Multiple answers possible)

- Basic and standard care
- Focused care
- Maximum care
- University care

**Part B: Intra-abdominal hypertension (IAH) and abdominal compartment syndrome (ACS)**

B.1 Do IAH or ACS play a role in your clinical practice?

- Never
- Seldom
- Regularly
- Often

B.2 Have you diagnosed IAH and ACS more frequently since 2010?

- Yes
- No

B.3

How often did you diagnose IAH in 2015?

- 0 times
- 1 to 10 times
- > 10 times

How often did you diagnose ACS in 2015?

- 0 times
- 1 to 5 times
- > 5 times

B.4 How do you diagnose IAH or ACS (mark each with an X).

IAH:

- Solely based on clinical symptoms
- Based on clinical symptoms PLUS intra-abdominal pressure measurement
- Solely based on intra-abdominal pressure measurement

ACS:

- Solely based on clinical symptoms
- Based on clinical symptoms PLUS intra-abdominal pressure measurement.
- Solely based on intra-abdominal pressure measurement

What clinical symptoms are included in your diagnosis?

(1) ________________

(2) ________________

(3) ________________

B.5 Are you measuring intra-abdominal pressure (IAD)?

- No
- Seldom
- Regularly
- Often

B.6 How many times did you perform a decompressive laparotomy for IAH/ ACS at your hospital in 2015? _______

What was the survival rate in your hospital in 2015 after an ACS diagnosis?

- With decompression: _______ (%)
- Without decompression: _______ (%)

In 2015, how many times did your hospital have to leave the abdomen temporarily open postoperatively? _______

ICU, Intensive care unit; NICU, Neonatological intensive care unit; PICU, pediatric intensive care unit
