# Supplementary material for: Awareness and diagnosis for intra-abdominal hypertension (IAH) and abdominal compartment syndrome (ACS) in neonatal (NICU) and pediatric intensive care units (PICU) – a follow-up multicenter survey
Source: BMC Pediatr. 2023 Feb 17;23:82. doi: 10.1186/s12887-023-03881-x (PMC9936744; doi:10.1186/s12887-023-03881-x)
Supplement: Supplementary file 2 — Additional file 2: Supplement II. Presentation of answers depending on the medical focus of the intensive care units (ICU). [file 12887_2023_3881_MOESM2_ESM.docx]

1. **2010 Survey**

|  | **Question** | **Exclusive NICU (%)** | **NICU rather than PICU (%)** | **PICU rather than NICU (%)** | **Exclusive PICU (%)** | **p- value** |
| --- | --- | --- | --- | --- | --- | --- |
| B.1 | Occurence and relevance of IAH/ACS in clinical practice   - Never - Seldom - Regularly - Often | 67 (18/27)  33 (9/27)  0  0 | 55 (45/82)  39 (32/82)  6 (5/82)  0 | 44 (8/18)  44 (8/18)  12 (2/18)  0 | 0  72 (5/7)  14 (1/7)  14 (1/7) | ***0,002*** |
| B.2 | not applicable (question was only asked in 2016) |  |  |  |  | . |
| B.3 | Frequency of diagnosed IAH at answering ICU’s (during the entire year before)   - 0 times IAH - to 10 times IAH - 10 times IAH   Frequency of diagnosed ACS at answering ICU’s (during the entire year before)   - 0 times ACS - 1 to 5 times ACS - > 5 times ACS | 81 (22/27)  19 (5/27)  0  85 (23/27)  15 (4/27)  0 | 64 (52/82)  30 (25/82)  6 (5/82)  72 (59/82)  27 (22/82)  1 (1/82) | 50 (9/18)  44 (8/18)  6 (1/18)  67 (12/18)  33 (6/18)  0 | 17 (1/6)  50 (3/6)  33 (2/6)  57 (4/7)  29 (2/7)  14 (1/7) | ***0,020***  0.137 |
| B.4 | Awareness and use of current WSACS definitions (multiple choice)   - IAH definition correctly chosen (increased IAP) - ACS definition correctly chosen (IAH + new organ dysfunction) | 0  15 (4/27) | 5 (4/82)  17 (14/82) | 6 (1/18)  28 (5/18) | 14 (1/7)  43 (3/7) | 0.538  0.319 |
|  | Clinical symptoms stated to be associated with increased IAP in children   - Respiratory symptoms - Cardiovascular symptoms - Renal symptoms - Gastrointestinal symptoms - Hepatobiliary symptoms | 21 (7/33)  18 (6/33)  15 (5/33)  39 (13/33)  0 | 23 (23/102)  18 (18/102)  22 (22/102)  33 (34/102)  1 (1/102) | 27 (8/30)  16 (5/30)  23 (7/30)  27 (8/30)  7 (2/30) | 12 (2/18)  23 (4/18)  17 (3/18)  34 (6/18)  6 (1/18) |  |
| B.5 | Share of respondents measuring the IAP regularly | 11 (3/27) | 20 (16/82) | 33 (6/18) | 43 (3/7) | 0.180 |
| B.6 | Share of respondents having performed decompressive laparotomy  Stated survival rate of ACS patients   - Surgically treated children - Non- surgically treated children | 4(1/27)  50  no specified | 23 (19/81)  94  74 | 22 (4/18)  70  63 | 67 (4/6)  67  59 | ***0.009***  0.178  0.484 |

Abb.: ACS, Abdominal compartment syndrome; IAH, intra-abdominal hypertension; IAP, intra-abdominal pressure; ICU, intensive care unit; NICU, neonatal intensive care unit (for premature and newborn infants (up to 28 days of life)); PICU, pediatric intensive care unit (for older children from infancy to adolescence (beyond the 28th day of life))

1. **2016 Survey**

|  | **Question** | **Exclusive NICU (%)** | **NICU rather than PICU (%)** | **PICU rather than NICU (%)** | **Exclusive PICU (%)** | **p- value** |
| --- | --- | --- | --- | --- | --- | --- |
| B.1 | Occurence and relevance of IAH/ACS in clinical practice   - Never - Seldom - Regularly - Often | 60 (24/40)  33 (13/40)  7 (3/40)  0 | 42 (36/86)  49 (42/86)  9 (8/86)  0 | 8 (1/13)  38 (5/13)  31 (4/13)  23 (3/13) | 12 (1/8)  63 (5/8)  25 (2/8)  0 | **<0.001** |
| B.2 | Increase in diagnosis of IAH and ACS since 2010 (requested exclusively in 2016) | 5 (2/39) | 12 (10/86) | 46 (6/13) | 37 (3/8) | **<0.001** |
| B.3 | Frequency of diagnosed IAH at answering ICU’s (during the entire year before)   - 0 times IAH - to 10 times IAH - 10 times IAH   Frequency of diagnosed ACS at answering ICU’s (during the entire year before)   - 0 times ACS - 1 to 5 times ACS - > 5 times ACS | 72 (28/39)  28 (11/39)  0  74 (28/38)  26 (10/38)  0 | 64 (54/84)  34 (29/84)  2 (1/84)  70 (58/83)  28 (23/83)  2 (2/83) | 25 (3/12)  50 (6/12)  25 (3/12)  18 (2/11)  64 (7/11)  18 (2/11) | 29 (2/7)  71 (5/7)  0  13 (1/8)  87 (7/8)  0 | **<0,001**  **<0.001** |
| B.4 | Awareness and use of current WSACS definitions (tested by multiple choice)   - IAH definition correctly chosen (increased IAP) - ACS definition correctly chosen (IAH + new organ dysfunction) | 0  42 (8/19) | 5 (3/61)  59 (34/59) | 18 (2/11)  85 (11/13) | 14 (1/7)  71 (5/7) | 0.190  0.990 |
|  | Clinical symptoms stated to be associated with increased IAP in children   - Respiratory symptoms - Cardiovascular symptoms - Renal symptoms - Gastrointestinal symptoms - Hepatic symptoms | 23 (12/52)  27 (14/52)  21 (11/52)  29 (15/52)  0 | 20 (34/169)  22 (38/169)  20 (33/169)  35 (59/169)  1 (2/169) | 32 (12/38)  13 (5/38)  21 (8/38)  29 (11/38)  0 | 18 (4/22)  9 (2/22)  27 (6/22)  36 (8/22)  9 (2/22) |  |
| B.5 | Share of respondents measuring the IAP | 26 (10/38) | 42 (36/85) | 92 (12/13) | 88 (7/8) | **<0.001** |
| B.6 | Share of respondents having performed decompressive laparotomy  Stated survival rate of ACS patients   - Surgically treated children - Non- surgically treated children   Share of respondents who needed to leave the abdomen open postoperatively (only 2016) | 20 (8/39)  86  24  23 (9/40) | 35 (30/85)  82  36  36 (31/86) | 70 (9/13)  85  59  85 (11/13) | 88 (7/8)  82  22  63 (5/8) | **<0.001**  0.572  0.405  **<0.001** |

ACS, Abdominal compartment syndrome; IAH, intra-abdominal hypertension; IAP, intra-abdominal pressure; ICU, intensive care unit; NICU, neonatal intensive care unit (for premature and newborn infants (up to 28 days of life)); PICU, pediatric intensive care unit (for older children from infancy to adolescence (beyond the 28th day of life))
