# Supplementary material for: Awareness and diagnosis for intra-abdominal hypertension (IAH) and abdominal compartment syndrome (ACS) in neonatal (NICU) and pediatric intensive care units (PICU) – a follow-up multicenter survey
Source: BMC Pediatr. 2023 Feb 17;23:82. doi: 10.1186/s12887-023-03881-x (PMC9936744; doi:10.1186/s12887-023-03881-x)
Supplement: Supplementary file 3 — Additional file 3: Supplement III. Presentation of answers depending on complete versus incomplete questionnaires (2016 survey). [file 12887_2023_3881_MOESM3_ESM.docx]

**Supplement III:** Presentation of answers depending on complete versus incomplete questionnaires (2016 survey)

|  | **Question** | **Complete questionnaires**  **53% (82/156)** | **Incomplete questionnaires 47% (74/156)** | **p- value** |
| --- | --- | --- | --- | --- |
| B.1 | Occurence and relevance of IAH/ACS in clinical practice   - Never - Seldom - Regularly - Often | 18 (15/82)  60 (49/82)  20 (16/82)  2 (2/82) | 74 (54/73)  22 (16/73)  3 (2/73)  1 (1/73) | **<0,001** |
| B.2 | Increase in diagnosis of IAH and ACS since 2010 (requested exclusively in 2016) | 21 (17/82) | 6 (4/72) | **0,006** |
| B.3 | Frequency of diagnosed IAH at answering ICU’s (during the entire year before)   - 0 times IAH - to 10 times IAH - 10 times IAH   Frequency of diagnosed ACS at answering ICU’s (during the entire year before)   - 0 times ACS - 1 to 5 times ACS - > 5 times ACS | 42 (34/82)  54 (44/82)  4 (3/82)  48 (39/82)  48 (39/82)  4 (4/82) | 87 (60/69)  12 (8/69)  1 (1/69)  86 (56/65)  14 (9/65)  0 | **<0,001**  **<0,001** |
| B.4 | Awareness and use of current WSACS definitions (tested by multiple choice)   - IAH definition correctly chosen (increased IAP) - ACS definition correctly chosen (increased IAP + new organ dysfunction) | 7 (6/82)  61 (50/82) | 0  47 (8/17) | 0,261  0,289 |
|  | Clinical symptoms stated to be associated with increased IAP in children   - Respiratory symptoms - Cardiovascular symptoms - Renal symptoms - Gastrointestinal symptoms - Hepatic symptoms | 21 (49/228)  21 (47/228)  22 (51/228)  34 (78/228)  1 (3/228) | 25 (14/55)  27 (15/55)  16 (9/55)  27 (15/55)  2 (1/55) |  |
| B.5 | Share of respondents measuring the IAP | 41 (34/82) | 14 (10/70) | **<0,001** |
| B.6 | Share of respondents having performed one decompressive laparotomy  Stated survival rate of ACS patients   - Surgically treated children - Non- surgically treated children   Share of respondents who need to leave the abdomen open postoperatively (only 2016) | 56 (45/82)  84  38  55 (45/82) | 14 (10/72)  88  47  16 (12/73) | **<0,001**  0.680  0.152  **<0,001** |

ACS, Abdominal compartment syndrome; IAH, intra-abdominal hypertension; IAP, intra-abdominal pressure; ICU, intensive care unit; NICU, neonatal intensive care unit (for premature and newborn infants (up to 28 days of life)); PICU, pediatric intensive care unit (for older children from infancy to adolescence (beyond the 28th day of life))
